# Glycosylation increases active site rigidity leading to improved enzyme stability and turnover

**DOI:** 10.1111/febs.16783

**Published:** 2023-04-03

**Authors:** Krithika Ramakrishnan, Rachel L. Johnson, Samuel D. Winter, Harley L. Worthy, Christopher Thomas, Diana C. Humer, Oliver Spadiut, Sarah H. Hindson, Stephen Wells, Andrew H. Barratt, Georgina E. Menzies, Christopher R. Pudney, D. Dafydd Jones

**Affiliations:** ^1^ Molecular Biosciences Division, School of Biosciences Cardiff University UK; ^2^ Department of Biology and Biochemistry University of Bath UK; ^3^ Biosciences, Faculty of Health and Life Sciences University of Exeter UK; ^4^ Ortho Clinical Diagnostics Pencoed UK; ^5^ Institute of Chemical, Environmental and Bioscience Engineering, Research Area Biochemical Engineering TU Wien Austria; ^6^ Department of Physics University of Bath UK; ^7^ Centre for Therapeutic Innovation University of Bath UK

**Keywords:** Enzyme enhancment, Enzyme rigidity, glycosylation, molecular dynamics, post‐translational modification

## Abstract

Glycosylation is the most prevalent protein post‐translational modification, with a quarter of glycosylated proteins having enzymatic properties. Yet, the full impact of glycosylation on the protein structure–function relationship, especially in enzymes, is still limited. Here, we show that glycosylation rigidifies the important commercial enzyme horseradish peroxidase (HRP), which in turn increases its turnover and stability. Circular dichroism spectroscopy revealed that glycosylation increased holo‐HRP's thermal stability and promoted significant helical structure in the absence of haem (apo‐HRP). Glycosylation also resulted in a 10‐fold increase in enzymatic turnover towards *o*‐phenylenediamine dihydrochloride when compared to its nonglycosylated form. Utilising a naturally occurring site‐specific probe of active site flexibility (Trp117) in combination with red‐edge excitation shift fluorescence spectroscopy, we found that glycosylation significantly rigidified the enzyme. *In silico* simulations confirmed that glycosylation largely decreased protein backbone flexibility, especially in regions close to the active site and the substrate access channel. Thus, our data show that glycosylation does not just have a passive effect on HRP stability but can exert long‐range effects that mediate the ‘native’ enzyme's activity and stability through changes in inherent dynamics.

## Graphical abstract

Glycosylation is one of the most common post‐translational modifications in nature. Yet, its impact on the enzyme structure–function relationship is limited. Using a combination of experimental and computational modelling, we found that the haem‐enzyme horseradish peroxidase becomes more rigid on glycosylation. Rather than increased rigidity impeding catalysis as per the classic ‘activity‐stability trade‐off’ idea, glycosylation increases turnover by 10‐fold and makes the enzyme more stable.

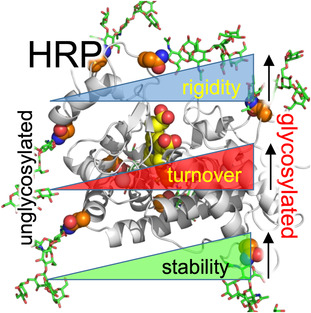

**Abbreviations**
CSM: centre of spectral mass
DAP: 2,3‐diaminophenazine
MD: molecular dynamics
OPD: *o*‐phenylenediamine
pHRP: plant horseradish peroxidase
PTM: post‐translational modification
REES: red‐edge excitation shift
rHRP: recombinant HRP
RMSD: root mean square deviation
RMSF: root mean square fluctuation

## Introduction

Post‐translational modification (PTM) of proteins is a common event in biology playing an essential role in imparting new structural and functional features on a protein [1]. Co‐factors such as haem expand the chemistry available to proteins allowing them to perform functions ranging from O_2_ transport to electron transfer to catalysis [2]. Glycosylation is the most prevalent PTM, with N‐linked glycosylation (via an asparagine residue in N‐X‐S/T sequence motif) the most common attachment mechanism [3, 4]. UniProt (www.uniprot.org/uniprotkb) reports that just over 87 000 proteins are glycosylated with *circa* 23 000 of those displaying catalytic activity. Given the number of glycosylated enzymes, it is important that we understand the impact of glycosylation on the enzyme structure–function relationship, especially if the enzymes are subsequently used for biotechnological applications and recombinant production is moved to different organisms.

While protein glycosylation is ubiquitous in eukaryotes, our knowledge of its impact on the protein structure–function relationship is still limited. Glycosylation plays several important biological roles [3] including protein quality control and molecular recognition. Glycan addition can also stabilise proteins by increasing solubility, promoting correct folding, making them more resistant to proteolysis and preventing aggregation [3, 5, 6, 7]. More recently, molecular dynamic (MD) simulations have suggested glycosylation can make a protein less flexible [8] despite the inherent flexibility of the glycans themselves [3, 9]. The question that arises is how glycosylation influences protein structure and thus in turn function, especially for enzymes where aspects such as dynamics can play a key role in defining catalysis [10, 11].

**Fig. 1:**
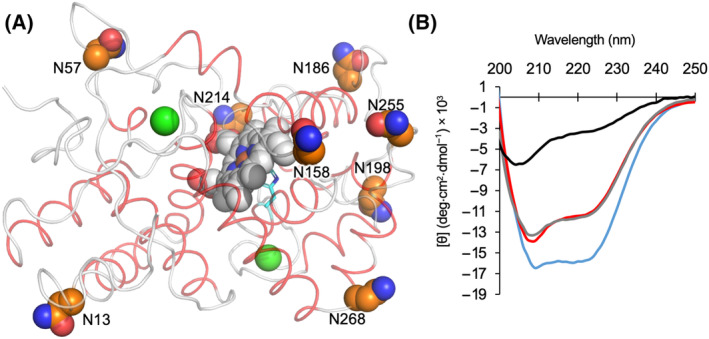
Structure of HRP. (A) Structure of HRP [19] (PDB 1HCH). Helical regions are coloured red, haem is shown as grey spheres, with the iron coordinating H170 shown as cyan coloured sticks and calcium ion as green spheres. The N‐linked glycosylated asparagine residues are shown as orange spheres. Figure was generated using pymol [70]. (B) Far‐UV CD spectra of holo and apo version of the glycosylated plant‐produced pHRP (blue and red, respectively) and the recombinantly produced holo‐ and apo‐rHRP (grey and black, respectively) at 25 °C.

Using horseradish peroxidase (HRP) as a model enzyme system, we probed experimentally how glycosylation together with co‐factor [haem] binding affects the dynamics and thus function and stability. HRP is an important commercial haem‐dependent peroxidase that has been extensively studied as a model for metal‐dependent oxidation reactions [12, 13, 14]. HRP is naturally derived from the roots of the Horseradish plant, *Armoracia rusticana*, with different isoforms produced. The most common isoform is C1A [15], which has in turn been the focus of recombinant expression [16, 17], structural studies [18, 19] and protein engineering [14, 20, 21]. HRP is monomeric and binds haem non‐covalently with His170 coordinating the haem iron ion (Fig. 1A). The naturally produced enzyme has eight N‐linked glycosylation sites [22, 23, 24, 25]. Recombinant versions produced in bacteria suggest that glycosylation is not absolutely essential to the folding or activity of the enzyme [16, 18]. Nevertheless, the recombinant expression does affect the physicochemical properties of HRP such as folding efficiency, solubility and aggregation propensity [26, 27, 28].

Here, we find that the glycosylated holo‐HRP is the most stable form of the enzyme and has a significantly higher catalytic turnover than the recombinant, non‐glycosylated holo‐HRP. Using a site‐specific fluorescence probe close to the active site (Trp117) naturally present in HRP, we experimentally find that the glycosylated holo‐HRP has the most rigid active site, but rigidification does not affect the temperature optimum for catalysis. MD and rigidity analysis reveals that glycosylation makes HRP less dynamic, especially around the active site. Thus, it appears that surface glycosylation not only globally stabilises HRP but exerts long‐range rigidifying effects into the core of the enzyme that influences catalysis.

## Results

### The effect of glycosylation and haem on HRP stability

Two sources of HRP were used here: directly from the plant (referred from herein as plant or pHRP) and a recombinant source produced in *Escherichia coli* (referred from herein as recombinant or rHRP). The rHRP is based on the C1A isoform and its production has been described previously [17]. The pHRP is commonly sourced and utilised by companies, including diagnostic companies; our source is clinical diagnostic grade pHRP used in tests manufactured by OrthoClinical Diagnostics. The pHRP is produced in the plant as the glycosylated holo‐form with haem bound. The apo‐form of pHRP is subsequently generated by means of a commonly used denaturation‐organic extraction process that removes noncovalently bound haem [29]. As we want to directly compare our pHRP with a defined recombinant HRP source, we used mass spectrometry to confirm the composition of both pHRP forms (Fig. S1 with supporting mass spectra analysis). Mass spectrometry confirms the number of N‐linked glycans present in pHRP to be 8. Apo‐ and holo‐ pHRP contain the same species that match the commonly observed C1A (with and without the C‐terminal serine) glycosylated forms [23, 30]. The absorbance spectra of pHRP and rHRP are very similar with a λ_max_ at 402–404 nm (Fig. S2). Thus, the main difference between pHRP and rHRP is the presence of N‐linked glycans so allowing us to investigate the effects of glycosylation on the enzyme and how glycosylation affects the structure and stability of the apo‐form.

We first looked at the impact of each PTM event on the general structure and stability of HRP using far‐UV circular dichroism (CD) spectroscopy. All HRP forms apart from apo‐rHRP show far‐UV CD spectra at 25 °C that are characteristic of predominately α‐helical structure reflective of the helical nature of HRP (Fig. 1B), exhibiting two signature minima at ~ 210 and ~ 220 nm (Fig. 1B). Glycosylated holo‐pHRP has deepest 210/222 nm troughs suggesting it has the highest helical content. The apo‐rHRP spectrum shows the protein is largely unfolded confirming the importance of haem to overall structural integrity. However, glycosylation can compensate for the loss of haem with the apo‐pHRP having a similar spectral profile to holo‐rHRP. These data therefore suggest that, at least in terms of the protein's secondary structure formation, HRP's global structure is influenced by both haem binding and glycosylation, with the latter promoting helical structure in the apo‐form of HRP, as well as the functional holo‐protein.

**Fig. 2:**
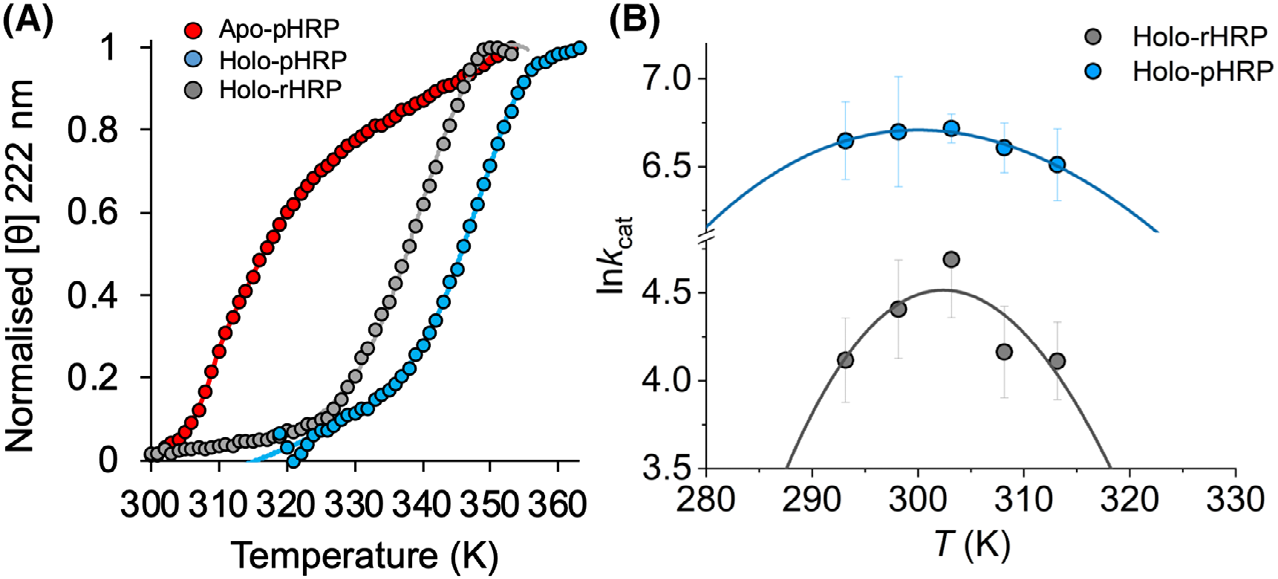
Temperature dependence of HRP structure and function. (A) Thermal melts measured by CD at 222 nm. The normalised molar ellipticity ([Θ]) was calculated by setting the value at 300 K to 0 and the value at 353 K (apo‐pHRP and holo‐rHRP) or 363 K (holo‐pHRP) to 1. The full thermal melt data is presented in Fig. S3, together with the data for apo‐rHRP that shows no clear transition. The lines represent a fit to Eqn (3) in the Materials and methods. (B) Temperature dependence of HRP turnover. Solid lines are the fits to Eqn (1). Apo‐pHRP is shown as red lines, holo‐pHRP as blue lines and holo‐rHRP as grey lines. Error bars represent standard deviations of the triplicate measurements.

Thermal unfolding was then measured to probe the effect of each PTM on stability. Apart from apo‐rHRP, the CD thermal melts (Fig. 2A) conform to apparent simple two‐state unfolding transitions. Our data suggest both haem binding and glycosylation stabilise HRP structure, with haem having the most significant effect. Holo‐pHRP is the most thermally stable with a *T*_m_ of 351 K, 9 K higher than holo‐rHRP (*T*_m_ 342 K). Apo‐pHRP melts at a lower temperature than both holoprotein forms (*T*_m_ 310 K), highlighting the integral nature of co‐factor binding to structural stability. Apo‐rHRP has a constant 222 nm signal with no clear transition confirming that it is largely unfolded prior to temperature ramping (Fig. S3). Thus, there is a clear stabilising effect of haem binding, translating to a > 30 °C stabilisation (in terms of *T*_m_). Glycosylation is also having a significant effect by increasing the stability of the holo‐protein and promoting the folding of the apo form, or at the very least promoting and/or stabilising helical elements. The effect of glycosylation is also consistent between the holo and apo enzyme, giving an increase in *T*_m_ on glycosylation of at least 9 °C. These observations point to a synergistic benefit of the combination of glycosylation and haem binding on HRP thermal stability.

### The effect of glycosylation on enzyme turnover

We next explored the effect of glycosylation on the steady‐state kinetics of HRP and the effect on the temperature dependence of HRP turnover by monitoring the conversion of the commonly used substrate *o*‐phenylenediamine (OPD) to the coloured 2,3‐diaminophenazine (DAP) [31, 32]. Table 1 (with Michaelis–Menten plots shown in Fig. S4) shows the Michaelis–Menten kinetics for holo‐rHRP and glycosylated holo‐pHRP at 298 K. The most significant effect of glycosylation is the large increase in turnover, with *k*_cat_ being an order of magnitude higher for holo‐pHRP *versus* holo‐rHRP. *K*_M_ remains relatively constant suggesting that it is catalysis rather than substrate binding being affected. Thus, glycosylation increases the rate by which HRP turns over OPD (Table 1). The temperature dependence of *k*_cat_ for both holo‐pHRP and holo‐rHRP show significant curvature with respect to temperature (Fig. 2B). Curvature in temperature dependence plots can arise from several sources, most commonly sub‐saturation of substrate binding, protein unfolding and change in the rate‐limiting step. In the absence of these factors, the curvature in temperature dependencies can reflect useful information on the thermodynamics of the chemical step and can be fit

(1) $$\ln k=\begin{array}{l} \ln\frac{k_{B}T}{h}-\left[\frac{{\Delta H}_{T_{0}}^{‡}+\Delta C_{P}^{‡}\left(T-T_{0}\right)}{\mathrm{RT}}\right]\\ +\;\left[\frac{{\Delta S}_{T_{0}}^{‡}+\Delta C_{P}^{‡}\left(\ln T-\ln T_{0}\right)}{R}\right], \end{array}$$

where *T*_0_ is an arbitrary reference temperature. $\Delta C_{P}^{‡}$ is the difference in heat capacity between the ground and transition states. $\Delta C_{P}^{‡}$ determines the change in Δ*H* ^‡^ and Δ*S* ^‡^ with temperature and thereby defines the nonlinearity of the temperature dependence of the Gibbs free energy difference between the ground state and the transition state (Δ*G* ^‡^).

**Table 1:** Thermodynamic and kinetic parameters of holo‐HRP.

	pHRP	rHRP
*T*_m_ (K)		
Holo	350.8 ± 2.7	342 ± 1.1
Apo	310.3 ± 0.3	NA^b^
*k*_cat_ (s^−1^)^a^	917.0 ± 53	86.2 ± 4.7
*K*_M_ (mm)^a^	0.92 ± 0.10	0.98 ± 0.13
*k*_cat_/*K*_M_ (s^−1^·m ^−1^)^a^	1.0 × 10^6^	8.82 × 10^4^
*T*_opt_ (K)	300.0	302.5
∆*H* ^‡^ (kJ·mol^−1^·K^−1^)^a^	1.3 ± 7.5	26.4 ± 8.5
∆*S* ^‡^ (kJ·mol^−1^·K^−1^)^a^	1.13 ± 0.05	1.18 ± 0.05
$\Delta C_{P}^{‡}$ (kJ·mol^−1^·K^−1^)	−1.9 ± 2.3	−6.6 ± 2.4

^a^ Reported for the production of DAP at 298 K from OPD. Curve fits are shown in Fig. S4.

^b^ Not applicable due protein being unfolded prior to temperature ramping.

We note our temperature‐dependent assays are performed with an excess of substrate (10× *K*_M_), and so, the curvature is not due to subsaturation of the *K*_M_ at elevated temperatures. Moreover, our steady‐state progress curves are entirely linear over the time course of the measurement, suggesting that there is no appreciable unfolding of HRP during the assay as the *T*_m_ values from CD thermal melts are ~ 30 °C higher than the highest temperature used in our kinetic studies. Therefore, fitting using the MMRT model appears appropriate. We cannot, however, explicitly rule out a change in rate‐limiting step with respect to temperature and so we focus our analysis on the apparent temperature optimum, rather than the microscopic interpretation of the magnitude of $\Delta C_{P}^{‡}$. The parameters resulting from fits to the temperature dependence of *k*_cat_ are given in Table 1; the only significant difference in the two HRP forms arises from the increased rate constant for pHRP over rHRP (Fig. 2A). Indeed, despite the measurable (Δ*T*_m_ ~ 9 °C; Table 1) difference in thermal stability and turnover number, the temperature optimum for enzyme turnover is essentially identical. That is, the temperature optimum for catalysis is not linked to the thermal stability of the enzyme.

### Using fluorescence to probe the effect of glycosylation on HRP rigidity

**Fig. 3:**
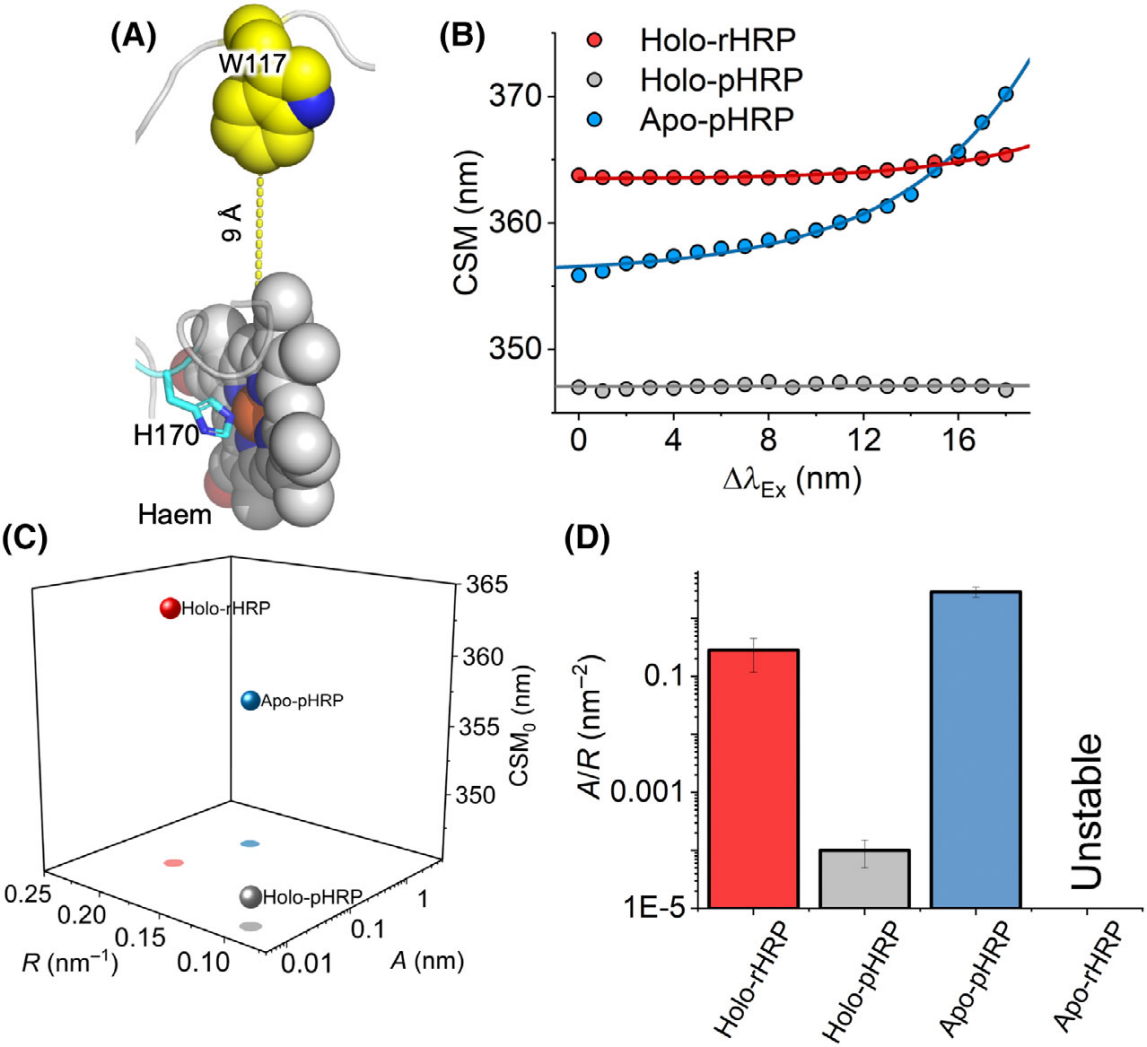
An active site‐specific REES probe captures shifts in flexibility. (A) Location of Trp117 relative to haem. Figure generated using pymol [70]. (B) Raw REES data, solid line shows the fit to Eqn (6) as described in the main text. (C) Plots of parameters resulting from the fits shown in panel B. (D) Ratio of parameters used to reflect shifts in molecular flexibility. Fluorescence emission spectra were measured at 298 K. Error bars represent standard error to the fit Eqn (2).

Given that both enzyme kinetics and thermal stability improved on glycosylation of HRP (Table 1), we experimentally investigated the local effect of glycosylation on active site rigidity/flexibility. As shown in Fig. 1A, all eight surface‐exposed N‐linked glycosylation sites are distant from the largely buried active site; the closest N‐linked site to the haem centre is N214 at ~ 20 Å. We have recently developed the use of an optical phenomenon that allows changes in the flexibility of a protein to be assessed via tryptophan fluorescence measurements: the red‐edge excitation shift (REES) [11, 33, 34, 35]. REES is an optical phenomenon, which tracks changes in the distribution of protein conformational states, via shifts in solvent‐fluorophore [Trp] interaction energies [36]. We find that the REES phenomenon, when quantified using our approach (described below), provides an extraordinarily sensitive metric of changes in flexibility. HRP affords an ideal, natural, site‐specific probe of the active site volume/rigidity as it has a single tryptophan residue (Trp117), 9 Å from the haem (edge‐to‐edge) (Fig. 3A). We have recently applied REES to engineered porphyrin binding proteins [37, 38] and were able to discriminate between differently flexible forms of a *de novo* designed artificial haem peroxidase that mapped precisely with NMR observations [38].

Figure 3 shows the REES data analysis for each of the HRP versions studied, except for apo‐rHRP; consistent with the findings from our CD data (Fig. 1B), apo‐rHRP showed a propensity to aggregate which significantly convoluted the fluorescence spectra. The REES data show an upward curvature in the magnitude of centre of spectral mass (CSM; Fig. 3B) with respect to change in emission wavelength (${\Delta \lambda }_{\mathrm{Ex}}$); for a single tryptophan‐containing protein, such curvature is indicative of a measurable REES effect and that the tryptophan is able to sample a range of different environments.

It is common to use $\lambda_{\mathrm{Em}}^{\max}$ of tryptophan fluorescence spectra to report on changes in the solvent exposure of tryptophan residues, with an increase in solvent exposure leading to an increase in $\lambda_{\mathrm{Em}}^{\max}$. The parameter CSM_0_ therefore reports similarly but is more accurate since it accounts for excitation energy‐dependent changes in fluorescence spectra. To quantify the REES phenomenon so as to compare changes in flexibility we fit the REES data to a simple exponential function

(2) $$\mathrm{CSM}={\mathrm{CSM}}_{0}+Ae^{R{\Delta \lambda }_{\mathrm{Ex}}}$$

where the amplitude and curvature of the exponential are described by *A* and *R*, respectively, and CSM_0_ is the CSM value independent of λ_Ex_. The plot of the resulting values from these fits is then shown in Fig. 2B. We have previously found that comparing the ratio of the *A* and *R* parameters is a simple way to infer changes in flexibility, with a large *A*/*R* value reflecting a more flexible protein/environment and smaller value reflecting a more rigid protein/environment [11, 33, 34, 39].

The data in Fig. 3 show that glycosylation (holo‐pHRP *versus* holo‐rHRP) results in a significant reduction in *A*/*R* and CSM_0_, respectively. That is, glycosylation of the protein surface alters the rigidity of the partially buried active site. On removal of the haem (apo‐pHRP), we find that the *A*/*R* increases dramatically, as well as showing an increase in CSM_0_ compared with holo‐pHRP; removal of the haem increases active site flexibility and solvent exposure. The influence of haem is not unexpected given it is buried within the protein and forms an integral structural component. The haem effect thus seems logical. The active site rigidification effect of glycosylation is unexpected as, compared with haem, the N‐linked sites are relatively distant from the Trp117 probe's site and surface‐exposed (Fig. 1A). Thus, the REES analysis suggests that the active site becomes more rigid on both glycosylation and haem binding, with holo‐pHRP being the most rigid.

### Molecular simulations of HRP dynamics

To further explore how glycosylation impacts on the dynamics of HRP, we undertook *in silico* simulations. There is currently no structure of the glycosylated HRP so glycan units were added *in silico* to each N‐linked site. Based on our mass spectrometry data (Fig. S1), the major holo‐HRP species contains eight N‐linked GlcNAc‐2, Man‐3, Fuc‐1 and Xyl‐1 glycan units [23, 30]. The glycan units, shown in Fig. S5, were added to the HRP structure using the PDB manipulation modules of the CHARMM‐GUI solution builder. MDs simulations for the glycosylated and nonglycosylated HRP (PDB entry 1HCH [19] modified for use with gromacs) were run for 100 ns (Fig. S6).

**Fig. 4:**
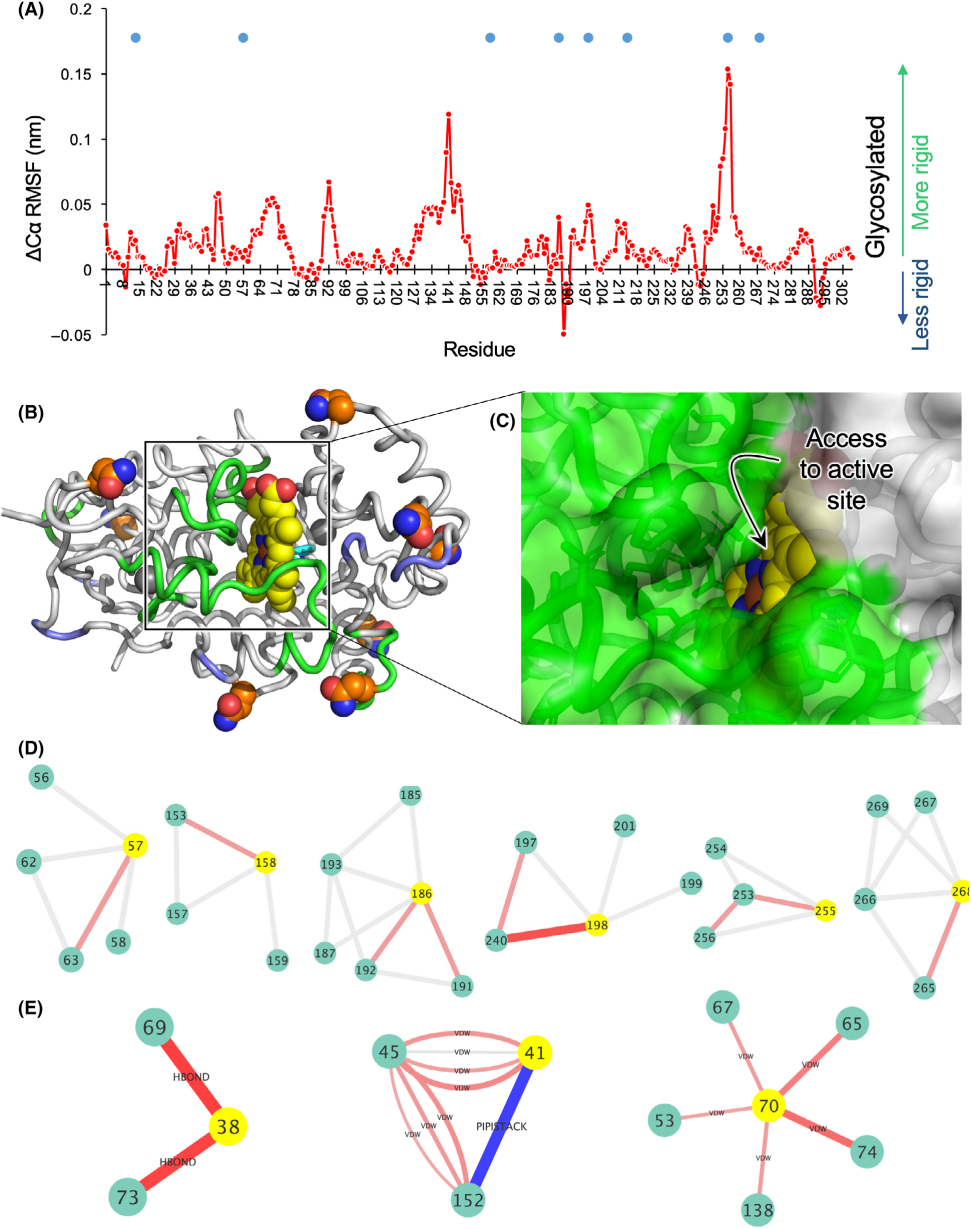
Molecular dynamic simulations of holo‐HRP. (A) Change in root mean squared fluctuation (RMSF) for residue's Cα over 100 ns. The blue circles represent the N‐linked glycosylation sites. The actual Cα RMSF values for each form are shown in Fig. S6b. The ΔCα was calculated by subtracting the RMSF value for the glycosylated holo‐HRP from the unglycosylated form. (B) Mapping the RMSF changes onto the structure of HRP. Green regions become less dynamic on glycosylation and blue regions become more dynamic. N‐linked sites are shown as orange spheres and haem as yellow spheres. (C) Surface representation of HRP showing the active site tunnel and access to the distal haem plane. Colours are as in (B). Figure B and C generated using pymol [70]. (D) Pairwise network for Cα atoms related to the N‐linked sites (shown as yellow spheres) over the course of the MD simulation. The full Cα pairwise network is shown in Fig. S7. Grey links represent no change in the Cα pairwise distances with red lines indicating closer distances with shade of red and thickness of the line related to the change in distance (ranging from light red thinner lines representing 0.38 Å to thicker darker red lines representing 0.76 Å). (E) Pairwise network of interactions for key catalytic residues (shown as yellow spheres) over the course of the simulation. Red lines indicate increased number of interactions in the glycosylated HRP and blue lines increased number of interactions in the nonglycosylated form. The thickness of the lines corresponds to the frequency of an interaction over the course of the MD, with thicker lines representing more persistent interactions. An arbitrary 10% cut‐off was applied, with interactions differences below this value ignored. The change in interaction type is shown on the diagram with VDW, HBOND and PIPISTACK equivalent to van der Waals, H‐bonds and pi‐pi stacking, respectively.

Glycosylation on the whole reduces Cα flexibility, which is indicative of increased backbone rigidity, with only a few residues where flexibility increased (Fig. 4A,B). Of 306 residues, 126 residues have a significantly (> 10% in the change in root mean square fluctuation's (ΔRMSF) maximal value) lower Cα RMSF on glycosylation while only 20 residues are significantly more flexible on glycosylation. Of the eight N‐linked asparagine residues, five show a significant reduction in RMSF (N13, N57, N186, N198, N255 and N268) when glycosylated, with none exhibiting an increase; N255 underwent the largest relative drop in glycosylation (1.53 Å). The haem group was relatively stable, with only 0.07 Å RMSF difference between the two HRP forms. The imidazole side chain of the haem coordinating residue H170 is less flexible (ΔRMSF 0.26 Å) in the glycosylated form. The central probe for REES, Trp117, does not change significantly on glycosylation (Fig. 4A) suggesting it is reporting on HRP dynamics as a whole rather than changes to the residue itself. MD also suggested that the pHRP has a higher proportion of helical content (46%) compared with rHRP (41%), a similar trend (albeit to a lesser extent) to that observed in the CD spectra (Fig. 1B).

Mapping the significant changes on Cα RMSF onto the structure of HRP reveals that many of the regions that exhibited reduced flexibility lie close to the active site entrance and directly interacting with the distal, catalytic plane of haem (Fig. 4B,C). Residues 68–72 together with residues 137–143 contribute towards forming the tunnel into the central iron ion of haem and the substrate binding site. The latter region includes the conserved P139‐A140‐P141 motif commonly found in plant peroxidases [13]. Residues 248–257 undergo the largest drop in Cα RMSF on glycosylation and are located proximal to the active site tunnel residues. The clustered model from the glycosylated MD run suggests that the glycan unit attached to N255 is potentially interacting with the N158 glycan unit. F41 together with H42 and its H‐bond coupled partner, N70, are critical to catalysis [40], with all three showing reduced Cα flexibility on glycosylation despite being buried and far from any of the N‐linked glycosylation sites. H42 in particular is critical to catalysis as it plays an essential role in the formation of compound I through accepting a proton from H_2_O_2_. The region on either side of M284, which makes direct contact with haem, also becomes less flexible on glycosylation.

We then undertook a pair‐wise network analysis to assess changes in the Cα distances over the course of the simulation, with a cut‐off distance of 5.5 Å. While little change was observed for most pairs, significantly more Cα pairs were closer together in the glycosylated (64/614) form compared with the non‐glycosylated form (26/614) (see Fig. S7 for full pair‐wise network). Moreover, a larger number of long‐range Cα pairwise distances (> *i*,*i* + 4) were closer in the glycosylated HRP (42%) compared with the non‐glycosylated enzyme (19%). Amongst the residues whose Cα become closer to their neighbours include key catalytic residues H170 (proximal haem iron coordination) and its associated residue N247 along with N70 (Fig. S8). With respect to the N‐linked site, six of the eight Asn Cα are closer to their neighbours on glycosylation (Fig. 4D); the remaining two N‐linked Asn sites (13 and 214) did not change significantly on glycosylation. This suggests that glycosylation is promoting a closer association with neighbouring residues.

We next looked at differences in the pairwise interactions over the course of the simulation. Similar to the Cα distances, the N‐linked Asn site generally increased the number of persistent interactions with neighbouring residues on glycosylation (Fig. S7). With regards to active site residues, the guanidino group of Arg38 increases its propensity to form H‐bonds with Gly69 and Ser73 (Fig. 4E). Asn70 likewise, increases its van der Waals interaction network with spatially local residues. Phe41 on the other hand has a lower propensity to form a pi‐pi stacking interaction with Phe152 on glycosylation. A pi‐pi interaction with Phe152 in the non‐glycosylated form could potentially disrupt the conformation of Phe41 relative to haem and thus may impact negatively on catalysis.

**Fig. 5:**
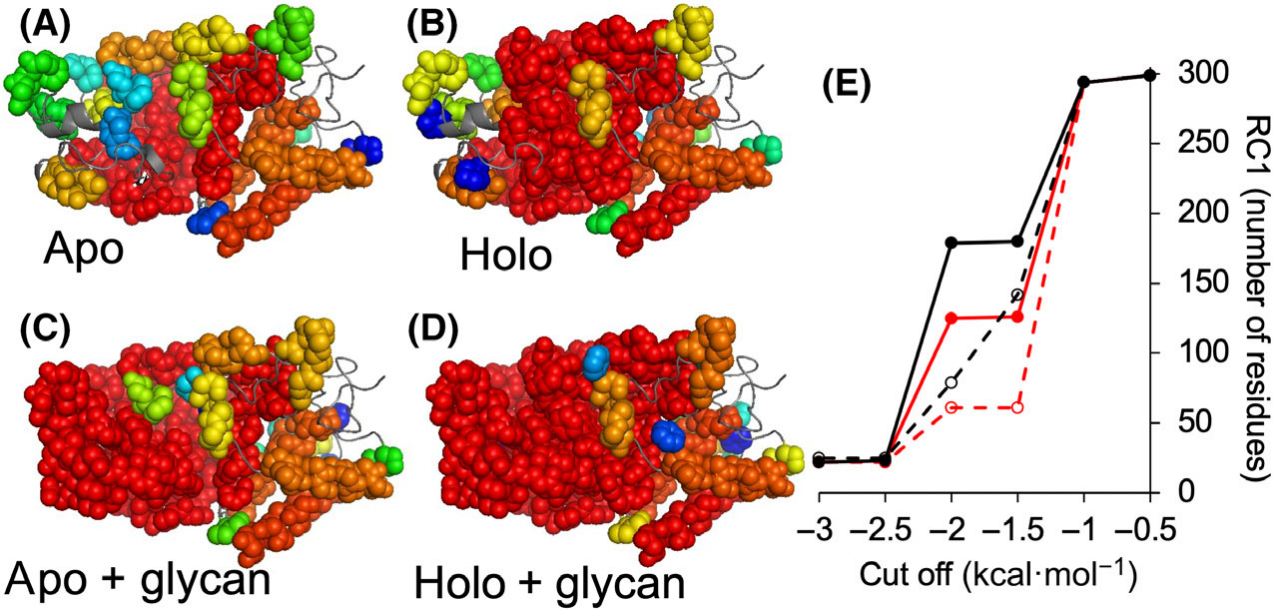
Rigidity analysis of the different HRP forms. (A–D) Structural representation of hydrogen‐bond energy cut‐off of −1.5 kcal·mol^−1^ for each form. The 20 largest rigid clusters are shown in space‐filling representation and rainbow‐coloured from red to blue. Flexible regions are shown as grey cartoon. Figures generated using pymol [70]. (E) Cluster size at each cut‐off point. RC1 value is the number of residues, which are members of the largest rigid cluster. Red and black represent unglycosylated and glycosylated forms, respectively, with dashed and full lines representing the apo‐ and holo‐forms, respectively. More details can be found in Table S1.

Structure‐based calculations can excel in detecting networks of rigid clusters through proteins and we have successfully used the FLEXOME implementation of pebble‐game rigidity analysis previously for this purpose [41, 42]. We initially performed a rigidity analysis on the available holo‐HRP structure [19]. The main change in rigidity occurs as the cut‐off is reduced from −1.0 to −3.0 kcal·mol^−1^ over steps of 0.5 kcal·mol^−1^ (Fig. 5A and Fig. S9). When the cut‐off is small, so that even relatively weak hydrogen bonds are included as constraints, a single large rigid cluster extends across almost the entire structure. As the cut‐off becomes more negative, excluding the weaker hydrogen bonds, peripheral portions of the structure become flexible, while the central part of the protein around the haem group retains rigidity. At the lowest cut‐offs explored here (−3.0 to −4.0 kcal·mol^−1^), the largest rigid cluster includes only about 20 residues around the haem group (Fig. 5E), representing the ‘rigid core’ of the protein.

As there are no structures available for the apo‐protein or glycosylated HRP, we used the available X‐ray crystal structure for holo‐rHRP to produce models. The apo‐HRP was generated by simply removing haem from the structure coordinate file prior to analysis. While our experimental evidence shows apo‐rHRP is unlikely to have any discernible structure, the cluster analysis does allow us to demonstrate the effect of haem removal on the native structure. Removal of haem has a substantial effect on the rigidity of the protein (Fig. 5B,E) as haem itself is a rigid body and forms a large number of noncovalent interactions, mostly hydrophobic interactions with surrounding residues. As a result, apo‐HRP loses its rigidity much more rapidly as the cut‐off becomes more negative. At a −1.5 kcal·mol^−1^ cut‐off, only 61 residues comprise the largest rigid cluster for apo‐HRP compared with 126 for holo‐HRP. This confirms experimental data regarding the importance of haem to maintaining the structural integrity of HRP.

To generate a rigidity mimic of the glycosylated protein, based on previous observations [43, 44, 45, 46] we assumed that attachment of a bulky glycan to the modified asparagine residues will reduce conformational flexibility in the vicinity of that residue, which is largely borne out in the MD simulations (Fig. 4A). We therefore introduced additional artificial constraints in the bond network between the Cα of the glycosylated residue and the Cα of the preceding and following residues in the sequence. The additional constraints are added to the bond list before rigidity analysis is carried out. These additional constraints rigidify both the apo‐ and holo‐forms with respect to non‐glycosylated form (Fig. 4). As in the case of the apo/holo comparison, the difference is observed in the intermediate cut‐off regime (−1.5 and −2.0 kcal·mol^−1^; Fig. 4E).

From Fig. 5, we find that rigidification due to glycosylation is visible in peripheral regions of the protein, whereas removal of the haem group has more effect in the central region around the haem binding pocket. That is, our rigidity analysis shows a logical finding that PTMs affect rigidity local to the site of the PTM. However, rigidity is not a strictly local phenomenon but rather percolates across constraint networks; we see in Fig. 5 the extent of rigidity in the glycosylated HRP structures, such that peripheral and central regions form a single rigid cluster, whereas in the non‐glycosylated HRP structures, the periphery is flexible relative to the central region. As a global view, we find that the trend in rigidity, based on the size of the largest rigid cluster in the intermediate cut‐off regime, is Holo‐pHRP > Holo‐rHRP > Apo‐pHRP > Apo‐rHRP. This trend exactly mirrors the CD stability data from the extracted *T*_m_ values (Table 1) and the REES data (Fig. 2), acknowledging the evident unfolding of Apo‐rHRP.

## Discussion

Post‐translational modification events such as glycosylation and co‐factor binding are important in biology by imparting essential structural and functional features on a protein. We show here that these two PTM events also influence protein dynamics leading to enhanced stability and, in the case of glycosylation, improved catalytic performance. Our data show a remarkable correlation between protein stability and active site rigidity. That is, the ranked rigidity of the active site from the Trp117 REES probe is Holo‐pHRP > Holo‐rHRP > Apo‐pHRP ≫ Apo‐rHRP, which mirrors the CD stability data. Our data suggest that glycosylated HRP is a better enzyme both in terms of stability and activity compared with the equivalent non‐glycosylated form, with both experimental and simulation data indicating the rigidification of the active site playing a major role.

Co‐factors such as haem commonly become integral structural components essential to protein structure and stability, as well as being necessary for function [2]. This holds true for HRP, with the haem co‐factor being buried within the enzyme's structure making extensive interactions with the protein. Removing haem therefore has an adverse effect on stability and structure as a whole, as our data clearly show (Figs 1, 2, 3). Glycosylation on the other hand occurs on the surface of a protein at multiple sites distant from the active site of enzymes, as illustrated here with HRP (Fig. 1A); the closest N‐linked site is ~ 20 Å away from the central iron atom of haem. Thus, the effect of glycosylation on HRP, especially the turnover enhancement, is not immediately obvious. We propose that glycosylation of HRP has a dual effect linked by a common mechanism: improved stability and activity through rigidification.

The role of glycosylation in protecting against events such as proteolysis and aggregation together with improving solubility is well‐established and is largely considered the main mechanism for maintaining a correctly folded and active protein [3]. Improved thermodynamic stabilisation, as observed here for HRP and for other glycoproteins (see Hebert *et al*. [44] and Sola and Griebenow [47] for an overview) is also an important contribution. Indeed, our results show that glycosylation can in part make up for the loss of haem by producing a more helical structured and stable apo‐HRP (Figs 2 and 3); this is despite none of the N‐linked sites in HRP residing in a helix and previous work suggesting glycosylation rarely induces helical structure [48]. Thus, glycosylation may have a chaperone effect assisting HRP folding; glycans assisting folding has been proposed previously [7]. Increased stability can be achieved by rigidification through lowering the free energy minimum of the folded protein. In a more rigid protein, numerous interactions can become more persistent, as is the case for holo‐pHRP (Fig. 4d,e), which leads to an increased enthalpic contribution to thermodynamic stability. Increased rigidity does appear to be the case here with glycosylated HRP as demonstrated initially through the REES effect (Fig. 3) and backed up with MD (Fig. 4).

While glycosylation stabilises HRP, another direct benefit of increased rigidity of glycosylated holo‐HRP is a 10‐fold enhancement of OPD turnover (Fig. 2 and Table 1). Enhancement of activity upon glycosylation has been observed for various enzymes, [5, 44] but little is known about how glycan addition at sites remote from the active site is influencing enzyme activity. Indeed, it has been observed previously for HRP when plant‐produced enzyme is compared with the recombinant form [16, 21, 28, 49] but with little attention paid and no rationale provided for such an observation. Thus, the main cause of this activity enhancement is largely unknown. One of the main ideas is that the general stabilisation of a protein leads to a higher population of correctly folded active enzymes on glycosylation therefore a higher observed activity. However, this is unlikely to be the single major factor for HRP as the unglycosylated protein is structurally stable over the optimal activity temperature (Fig. 2A) and has a turnover temperature optimum like that of the glycosylated form (Fig. 2B). It may well be that increased temperature stability is a consequence of increased rigidity. Moreover, while *k*_cat_ changes *K*_M_ is similar for both holo‐HRP forms suggesting changes to the catalytic process or the population of HRP capable of undertaking catalysis at any specific time point. Both our experimental (Fig. 3) and simulation (Figs 4 and 5) data suggest that enhanced activity is likely to be driven by propagated dynamical changes on glycosylation that lead to the rigidification of the active site.

Active site dynamics have emerged as having a significant effect on enzyme turnover and particularly manifesting in a measurable heat capacity of catalysis [10, 50]. Relevant to this study, recent work has shown that chemically induced structural rigidification of an artificial haem peroxidase leads to increased helical content, stability and turnover [38]. Unlike chemically induced rigidification, glycosylation is highly specific in terms of the structural regions directly impacted. Indeed, the site of N‐linked glycosylation is as critical as the glycan addition [44]; glycosylation at engineered non‐native sites will not automatically lead to enhanced stability and function [51, 52]. Additionally, significant local rigidification of the N‐linked site on glycosylation does not appear to be universal for HRP according to our MD simulations (Fig. 4A). Protein engineering has shown that mutational events can change conformational dynamics that in turn shape enzyme activity [39, 53, 54, 55]. But this is different from glycosylation as protein engineering changes the inherent amino acid sequence itself, commonly through mutations in or close to the active site, not the intrinsic dynamics of the existing protein sequence. Also, given the significant proportion of secreted proteins that are enzymes, glycosylation does appear to be a common mechanism nature uses to mediate enzyme activity.

So how does glycosylation at surface residues distant from the HRP active site impart their effect on activity? Given that the REES probe, Trp117, is close to the active site (Fig. 3A), we can assume that the active site becomes more rigid. The experimental data are backed up by the MD simulations (Fig. 4) and rigidity analysis (Fig. 5), with the former showing clearly that residues comprising the active site pocket and substrate tunnel being more rigid in the glycosylated form. Moreover, pairwise analysis shows that Cα is more often closer together in the glycosylated form, with key catalytic residues forming more persistent interactions (Fig. 5D,E). Glycosylation may also cause less favourable interactions that may impact on catalysis to become less persistent, such as the pi‐stacking interaction between Phe41 and Phe152 (Fig. 4E). MD also suggests it may not always be the nearest N‐linked glycosylation site in terms of the amino acid sequence that exerts the greatest effect; the rigidification of the active site pocket (residues 130–148) is likely to be derived from long‐range interaction changes associated with N‐linked glycosylation of N255, the residue that undergoes the largest reduction in Cα flexibility on glycosylation (Fig. 4A). Thus, significant local rigidification of selected N‐linked sites and associated propagated network changes may be enough for both enhanced function and thermal stability. Future work that involves systematic mutation of each N‐linked site followed by native glycosylation in a plant system could address this but is currently technically quite challenging given *A. rusticana* would need to be the ideal production host to preserve evolutionary‐linked glycosylation patterns and isoform production. What it does show is that currently the native enzyme from *A. rusticana* is the best source of HRP in terms of stability and activity towards OPD compared with its sequence equivalent recombinant enzyme. However, given that our work suggests that HRP activity and stability are linked to inherent enzyme rigidity, protein engineering could be used to accomplish the same task, with some notable successes for HRP [21, 49, 56, 57, 58] and other enzymes [59, 60] providing inspiration. Given the relative remoteness of surface‐exposed glycosylation sites relative to the active site, protein engineering could switch focus away from generally conserved active site residues to targeting peripheral residues to rigidify a protein, as has been shown in other enzyme systems [59, 60, 61].

To conclude, glycosylation of HRP does not simply appear to be an inert modification protecting the enzyme from aggregation but affects global protein stability and flexibility to the level of altering active site rigidity and thus function. Rigidification of HRP is correlated with a 10‐fold increase in turnover, but does not affect the temperature optimum for catalysis. Thus, the temperature optimum of catalysis and overall temperature stability of HRP are not necessarily directly correlated, coupled or the same. Indeed, thermal stability may simply be a beneficial by‐product of increased rigidity rather than the driving force for improved activity, as our work presented here and that of others (see Refs [10, 50] for an overview) are counter to the classical ‘stability‐activity trade‐off’ scenario. In our case, the most stable and rigid form of HRP is the most active at a given, relatively low temperature. Glycosylation thus provides HRP with both a functional and stability advantage.

## Materials and methods

### Protein production

Lyophilised glycosylated apo and holo plant HRP was obtained from Ortho Clinical Diagnostics (Pencoed, UK). Haem occupancy was confirmed spectrophotometrically yielding a Reinheitszahl constant (RZ *A*_404_/*A*_280_) 2.98 for holo‐protein (Fig. S2). Apo‐pHRP was as generated by Biozyme for Ortho Clinical Diagnostics and manufactured using a butanone extraction approach [29] before buffer exchange into water and lyophilisation. The composition of the pHRP protein was determined by ESI‐ToF Mass Spectrometry performed by the Analytical Services facility within the School of Chemistry, Cardiff University. Recombinant holo‐HRP was produced as described previously [17]. Haem occupancy was confirmed spectrophotometrically yielding a Reinheitszahl constant (RZ *A*_404_/*A*_280_) 2.91 (Fig. S2). Haem was removed using a butanone extraction approach as described previously [29].

### Circular dichroism

Circular dichroism measurements were performed on a Chirascan™ CD spectrometer (Applied Photophysics, Leatherhead, Surry, UK) using 5–10 μm protein in either 20 mm Bis‐Tris (pH 7.0) for rHRP or 50 mm Tris (pH 8.0) for pHRP. Spectra were recorded from 200 to 260 nm at 1 nm intervals. Samples were subsequently heated at a ramp rate of 1 °C·min^−1^ using a Quantum Northwest Peltier (Quantum Northwest, Liberty Lake, WA, USA) with absorbance at 222 nm at every degree change up to 90 °C. The CD signal at 222 nm was fit to Eqn (1) for a simple two‐state transition:

(3) $$\theta_{222\;\mathrm{nm}}=\frac{b_{f}+m_{f}T+\left(b_{u}+m_{u}T\right)K_{u}}{1+K_{u}},$$

where

(4) $$K_{u}=\exp\left(\Delta H\left(1-T/T_{m}\right)/\mathrm{RT}\right)$$

where *m* and *b* are the slope and intercept of the folded (f) and unfolded (u) baseline, respectively. *T*_m_ is the melting temperature and Δ*H* is the Van't Hoff enthalpy of unfolding at *T*_m_.

### Steady‐state kinetics

Steady‐state kinetics of HRP was carried out using the substrate *o*‐phenylenediamine dihydrochloride (OPD) (Sigma Aldrich, Gillingham, Dorset, UK) that is converted to the coloured product 2,3‐diaminophenazine (DAP) [31, 32]. For rHRP, the reaction conditions were 20 mm Bis‐Tris (pH 7.0), 3% v/v hydrogen peroxide and 10 nm enzyme. For pHRP the reaction conditions were 50 mm Tris (pH 8.0), 3% v/v hydrogen peroxide and 0.5 nm enzyme. Protein concentrations were calculated using 403 nm absorbance (ε = 100 mm ^−1^·cm^−1^) so only the concentration of holoprotein is considered. Freshly prepared OPD substrate ranging in concentration from 0.1 to 4.0 mm was added to the reaction mix. Absorbance was recorded at 450 nm over 60 s on a Cary UV Spectrophotometer (Aligilent, Santa Clara, CA, USA), in triplets for each substrate concentration. The substrate turnover per minute was calculated from the absorbance change over 1 min using the extinction coefficient of DAP product at 450 nm (10 600 m ^−1^·cm^−1^). The kinetic data were fitted to a Michaelis–Menten equation using graphpad prism software (Dotmatics, Boston, MA, USA) to determine *V*_max_, *K*_M_ and *k*_cat_. Reaction rates of pHRP in 20 mm Bis‐Tris (pH 7.0) were comparable to those in 50 mm Tris (pH 8.0). However, rHRP rates were lower in 50 mm Tris (pH 8.0) thus we used the more optimal 20 mm Bis‐Tris (pH 7.0) buffering conditions to measure rHRP kinetics.

Temperature‐dependent kinetics data were also obtained on a Cary UV Spectrophotometer where all reaction components were incubated at temperatures ranging from 293 K (20 °C) to 313 K (40 °C) for 1 h before mixing the components and recording the absorbance at 450 nm. The substrate (OPD) concentration used was equal to 10× of *K*_M_ value and absorbance change was recorded at 450 nm for 2 min, in triplicates for each temperature. Substrate turnover per minute was calculated as previously described using the extinction coefficient of DAP product.

### Red‐edge emission shift

Red‐edge emission shift (REES) of HRP was determined by measurement of fluorescence emission using 5 μm protein samples, which were prepared under the buffer conditions used for the steady‐state kinetics. Proteins were excited at 1 nm intervals over the range 292–310 nm and emission was recorded between 315 and 500 nm at a scan rate of 30 nm·min^−1^. For all readings, excitation and emission slit width was set to 5 nm with a detector voltage set to high. Up to 12 independent readings at each excitation wavelength were taken and the average was calculated. Haem is a known quench tryptophan fluorescence [62], which resulted in emission intensities for holo‐HRP being ~ 10% of that observed for the apo‐HRP.

The quantification of the REES data relies on the accurate extraction of information on how the structure of the emission spectra varies, and so, the additional band would convolve the measurement. As in our previous work [33, 35, 38, 63], we therefore numerically modelled each of the spectra using a sum of two skewed Gaussians (Eqn 5) as described recently for a *de novo* haem peroxidase [38]

(5) $$f_{i}=f_{\max}\exp\left(-\ln2\right){\left(\frac{\ln\left(1+\frac{2b\left(\lambda_{\mathrm{Em}}-\lambda_{\mathrm{Em}}^{\max}\right)}{w}\right)}{b}\right)}^{2},$$

where *f*_i_ is the measured fluorescence intensity, *f*_max_ is the maximum emission intensity at wavelength $\lambda_{\mathrm{Em}}^{\max}$, with a full width at half maximal of *w*, and the ‘skewness’ was controlled by *b*. Fluorescence spectra were accurately modelled and deconvolved by fitting to such functions as demonstrated elsewhere [33, 35, 38, 63]. By fitting to a sum of two skewed Gaussians we were able to accurately model the spectral component attributable to tryptophan emission alone. From these models, the centre of spectral mass (CSM) was extracted for each spectral component, which allowed the quantification of changes in the structure of the fluorescence spectra,

(6) $$\mathrm{CSM}=\frac{\sum \left(f_{i}\times \lambda_{\mathrm{Ex}}\right)}{\sum \left(f_{i}\right)}$$

The resulting plot of CSM versus the change in excitation wavelength, ${\Delta \lambda }_{\mathrm{Ex}}$, is shown in Fig. 3B for each of the HRP versions.

### Molecular dynamics

The PDB model for HRP (1H58) was uploaded to the solution builder page on the CHARMM‐GUI server ([64], https://www.charmm‐gui.org/?doc=input/solution) transferring the coordinates for the protein, heme residue and calcium ions. At the protein modification stage, default disulfide bonds were retained, and a heme coordination site was added at residue H170. To generate the glycosylated form of the protein, (Xyl)Man3(Fuc)GlcNac2—N‐1 (Fig. S5) was added to asparagine residues: N13, N57, N158, N186, N198, N214, N255 and N268 at the PDB manipulation stage of the CHARMM‐GUI solution builder. The simulations were set up in a cubic box with a 10 nm edge distance. Protein charge was neutralised by the addition of 150 mm CaCl_2_ ions and solvated. PME FFT grid was generated automatically by the server. Parameters and input files for use in gromacs using the CHARMM36m forcefield were then generated with hydrogen mass repartitioning and WYF cation‐pi interactions [65, 66]. Input files were downloaded from the server to run MD. MD was run through three stages, minimisation, equilibration and production. Minimisation used the steepest decent method with a tolerance of 1000 kJ^−1^·nm^−1^ and a cut‐off of 5000 steps. The equilibrium stage was run for 125 000 steps with a 0.001 fs time per step. Finally, the production run was carried out for 100 ns total time with a 0.004 fs time per step. A temperature of 303.15 °K, a pressure of 1 atm and periodic boundary conditions were applied to all simulations. For all simulations, Nose‐Hoover temperature coupling thermostat was applied and Particle‐mesh Ewald (PME) was applied to long‐range electrostatics. Analysis was carried out on both production runs using inbuilt gromacs modules.

### Pairwise network analysis

The Cα‐Cα distance changes were determined as follows. Using the gromacs mdmat command, a distance matrix was generated for both nonglycosylated and glycosylated MD simulations, which consisted of the smallest distance between each residue over the course of each simulation. This distance matrix was converted from xpm to csv format using a python script. Both distance matrices were then parsed using a custom jupyter notebook and merged to calculate the change in Cα‐Cα distance. A function was written to only consider the change in Cα‐Cα distances between residues that were within 5.5 Å. Another function was written to only consider Cα‐Cα distances between residues that were greater than *i* + 4 apart. The resulting adjacency list was analysed and visualised as a network using cytoscape 3.9.1 [67]. The interaction networks were determined as follows. Using pymol, gromacs trajectories were converted to PDB ensembles. Resulting PDB ensembles were uploaded to the ring 3.0 [68] server to identify noncovalent interactions and their frequency over the duration of the simulation. ‘Strict’ distance thresholds were used to classify inter‐residue interactions. The resulting machine‐readable JSON files were converted to csv using cytoscape. Both csv files were then merged using a custom jupyter notebook where a function was written to only extract interaction frequencies that underwent a change +/− 10%. The resulting csv file was imported to cytoscape for analysis and network visualisation. All scripts and notebooks used in this analysis are available at https://github.com/DrewBarratt/MD_Network_analysis

### Rigidity analysis

Pebble‐game rigidity analysis [42, 69] is an integer algorithm, which can divide a protein structure into rigid clusters and flexible regions by matching degrees of freedom against bonding constraints on a directed graph constructed from the covalent and noncovalent interactions of the protein. Glycosylation was modelled by the addition of artificial constraints to the covalent interaction network, reducing the conformational flexibility of the backbone at glycosylation sites. The results of this division, or rigid cluster decomposition (RCD), depend on which noncovalent interactions are included and are therefore a function of a hydrogen‐bond energy cut‐off, which excludes weaker polar interactions such as hydrogen bonds. A ‘rigidity dilution’ is carried out by progressively altering this cut‐off from small negative values to larger negative values, gradually excluding polar interactions from weaker to stronger. The most rigid portions of the structure can be identified as those which retain rigidity longest during this dilution. The analysis was carried out on an all‐atom model of the protein and rigid clusters can extend across main‐chain and side‐chain structural groups. For this study, a residue is considered part of the largest rigid cluster (RC1) if its Cα is part of that cluster. The rigidity analysis software used here (‘FLEXOME’, written by SAW) is available on request from the University of Bath (https://doi.org/10.15125/BATH‐00940).

## Conflict of interest

The authors declare no conflict of interest.

## Author contributions

KR and RLJ collected and analysed the data associated with enzyme kinetics, CD and REES. SDW contributed to the collection and analysis of CD data. HLW and GEM run and analysed molecular dynamics. CT contributed to pHRP samples. DCH and OS contributed to rHRP samples. SHH contributed to REES and CD analysis. SW performed and analysed the rigidity analysis. AHB generated and analysed the pairwise analysis data. CRP contributed to extended enzyme kinetic and REES analysis, conceived the idea and help manage the project. DDJ conceived the idea, managed the project and contributed to the analysis of the enzyme kinetics, CD, MD and pairwise analysis. All authors contributed to the writing of the manuscript.

### Peer Review

The peer review history for this article is available at https://www.webofscience.com/api/gateway/wos/peer‐review/10.1111/febs.16783.

## Supporting information


**Fig. S1.** Analysis of the plant HRP.
**Fig. S2.** Absorbance spectra of holo‐pHRP (green) and holo‐rHRP (black).
**Fig. S3.** CD analysis of HRP. Thermal melt data as measured by CD at 222 nm for each form of HRP.
**Fig. S4.** Steady‐state enzyme kinetics of (a) holo‐pHRP and (b) holo‐rHRP.
**Fig. S5.** Glycosylated model of HRP.
**Fig. S6.** Molecular dynamics of glycosylated (red) and nonglycosylated (black) HRP.
**Fig. S7.** Pairwise network of Ca atoms with a 5.5 Å cut‐off over the course of the MD simulation.
**Fig. S8.** Change in pairwise interactions related to N‐linked glycosylated Asn residues (yellow spheres) over the course of the MD simulation.
**Fig. S9.** Rigid cluster decompositions (RCDs) of holo‐HRP at different hydrogen‐bond energy cut‐offs in the course of a rigidity dilution.
**Table S1.** Rigidity analysis of the different HRP forms.

## Data Availability

Data and analysis approaches are available either from web links within the manuscript or from the repository at Cardiff (https://doi.org/10.17035/d.2022.0215403417).
