# Supplementary material for: Glycosylation increases active site rigidity leading to improved enzyme stability and turnover
Source: FEBS J. 2023 Apr 3;290(15):3812–27. doi: 10.1111/febs.16783 (PMC10952495; doi:10.1111/febs.16783)
Supplement: Supplementary file 1 — Fig. S1. Analysis of the plant HRP. Fig. S2. Absorbance spectra of holo‐pHRP (green) and holo‐rHRP (black). Fig. S3. CD analysis of HRP. Thermal melt data as measured by CD at 222 nm for each form of HRP. Fig. S4. Steady‐state enzyme kinetics of (a) holo‐pHRP and (b) holo‐rHRP. Fig. S5. Glycosylated model of HRP. Fig. S6. Molecular dynamics of glycosylated (red) and nonglycosylated (black) HRP. Fig. S7. Pairwise network of Ca atoms with a 5.5 Å cut‐off over the course of the MD simulation. Fig. S8. Change in pairwise interactions related to N‐linked glycosylated Asn residues (yellow spheres) over the course of the MD simulation. Fig. S9. Rigid cluster decompositions (RCDs) of holo‐HRP at different hydrogen‐bond energy cut‐offs in the course of a rigidity dilution. Table S1. Rigidity analysis of the different HRP forms. [file FEBS-290-3812-s001.pdf]

Supporting Information.

Glycosylation increases active site rigidity leading to improved enzyme stability and turnover

Krithika Ramakrishnan, Rachel L. Johnson, Samuel D. Winter, Harley L. Worthy, Christopher Thomas, Diana C.Humer, Oliver Spadiut, Sarah H. Hindson, Stephen Wells, Andrew H. Barratt, Georgina E. Menzies, Christopher R. Pudney and D. Dafydd Jones **

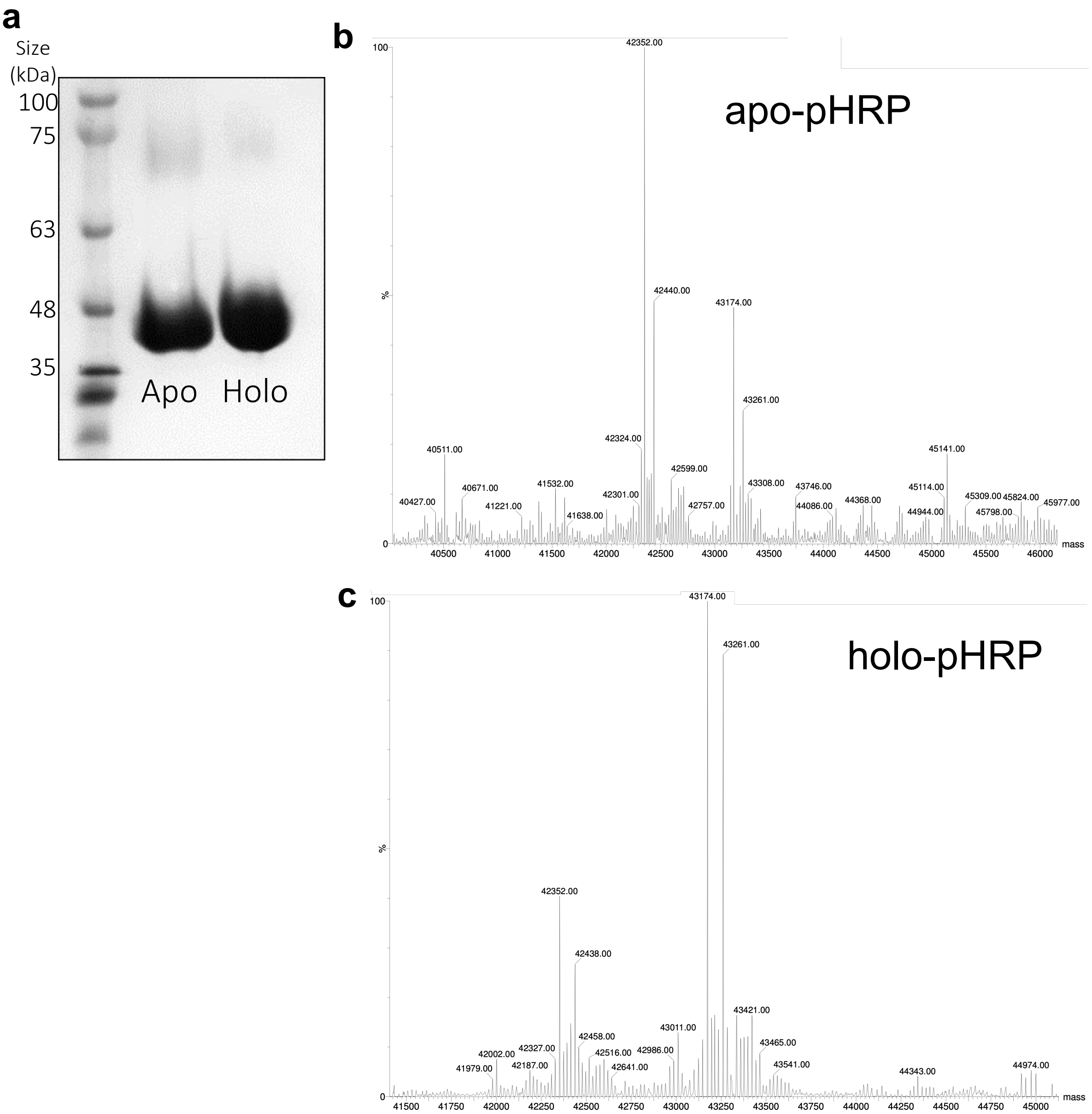

Figure S1. Analysis of the plant HRP. (a) SDS PAGE and (b-c) mass spectrometry of (a, b) apo and (a, c) holo plant HRP. **Supplementary analysis.** The pHRP samples are directly used in immunoassays manufactured by OrthoClinical Diagnostics (OCD). The apo-rHRP is generated by an organic extraction process by the supplier and used as a correction agent in certain OCD diagnostic immunoassays. SDS PAGE confirms the samples' purity. Mass spectrometry was used to analyse the composition of both the apo (b) and holo (c) versions. Previous studies have shown that the main polypeptide unit is comprised of Gln31-Ser338, which has a theoretical mass of 33918 Da [1, 2]. Both samples show two main peaks at ~42kDa (42352 Da and 42440 Da) and ~43 kDa (43174 Da and 43261 Da), with each divided into 2 sub peaks. The masses match previously analysed plant HRP samples [3]. The difference in mass between the two sub peaks is 87-88 Da, which is equivalent to the processing of the C-terminal serine, a common feature of the C1A isoform [3-5]. The two main glycan forms have an estimated masses of 8530 Da and 9351 Da. They represent 7 N-linked carbohydrate units (GlcNAc-2, Man-3, Fuc-1, Xyl-1) with one disaccharide (GlcNAc-1, Fuc-1) and 8 N-linked carbohydrate units (GlcNAc-2, Man-3, Fuc-1, Xyl-1), respectively,

as reported previously [3, 5]. Both apo and holo forms of pHRP contain the same components but with the apo-form having an apparent higher proportion of the single disaccharide glycan form. The haem is non-covalently bound so will be lost during the measurement process; thus both measured masses will be equivalent to the apo-protein mass.

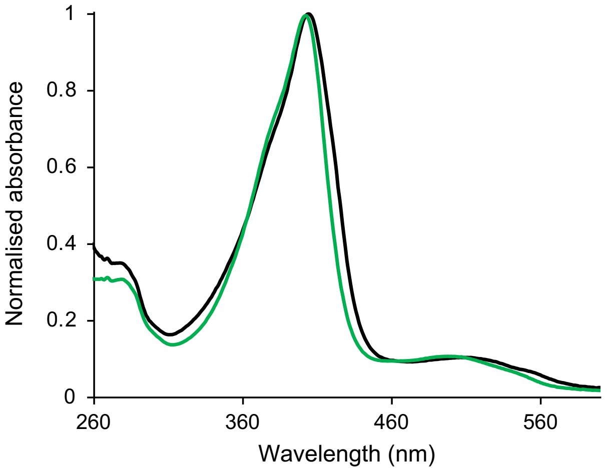

Figure S2. Absorbance spectra of holo-pHRP (green) and holo-rHRP (black).

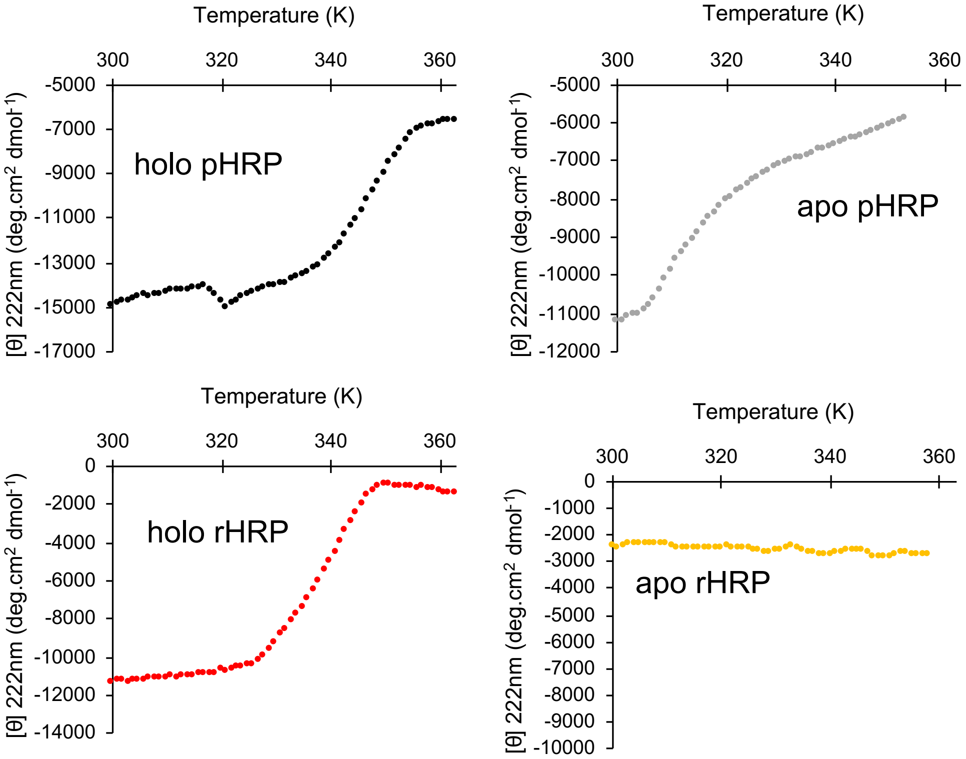

Figure S3. CD analysis of HRP. Thermal melt data as measured by CD at 222nm for each form of HRP.

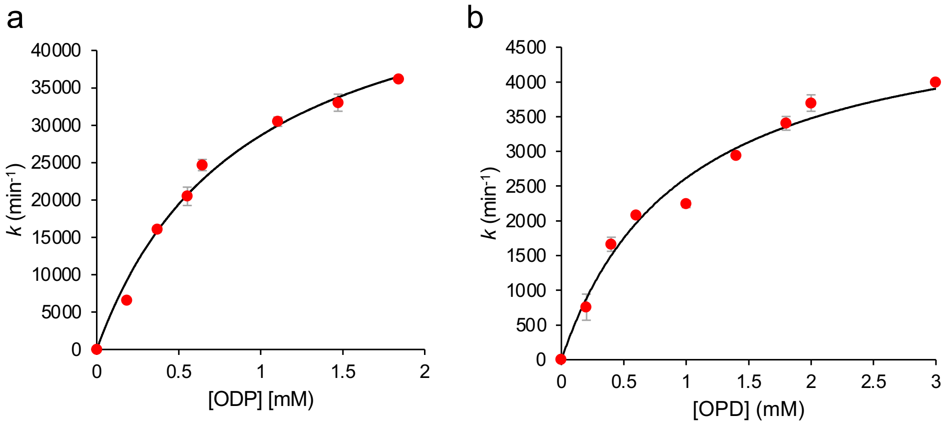

Figure S4. Steady state enzyme kinetics of (a) holo-pHRP and (b) holo-rHRP. The rate was determined by monitoring the production of DAP at 450 nm from OPD. The rates where fit to the Michaelis Menten equation. Error bars are the standard deviation of triplicate measurements.

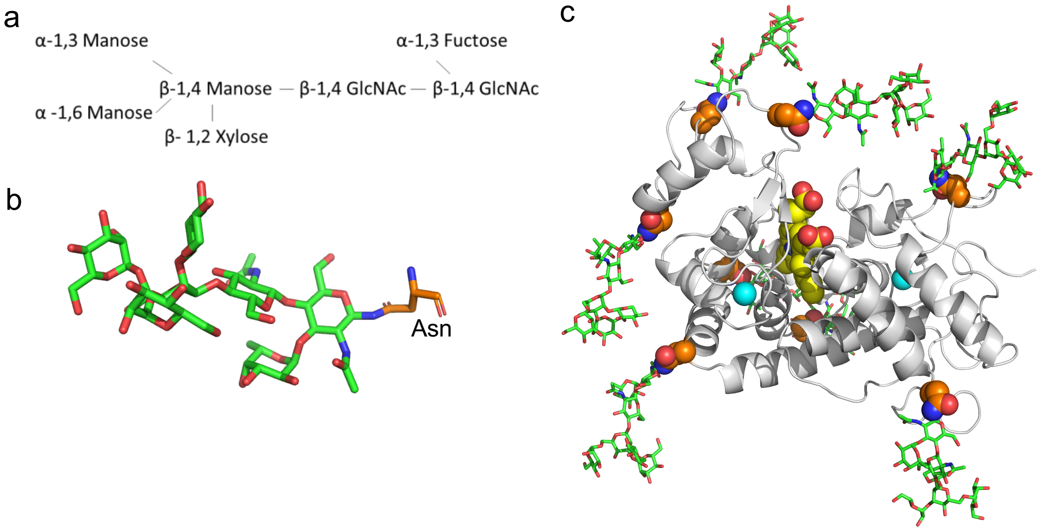

Figure S5. Glycosylated model of HRP. (a) Outline of the glycan composition and connectivity together with (b) a representative N-linked unit bound used in the simulation. (c) Clustered average of the glycosylated HRP model with the glycan units coloured green, the asparagine residues orange, calcium cyan spheres, and haem as yellow spheres. Figures 5b-c generated using PyMOL [6].

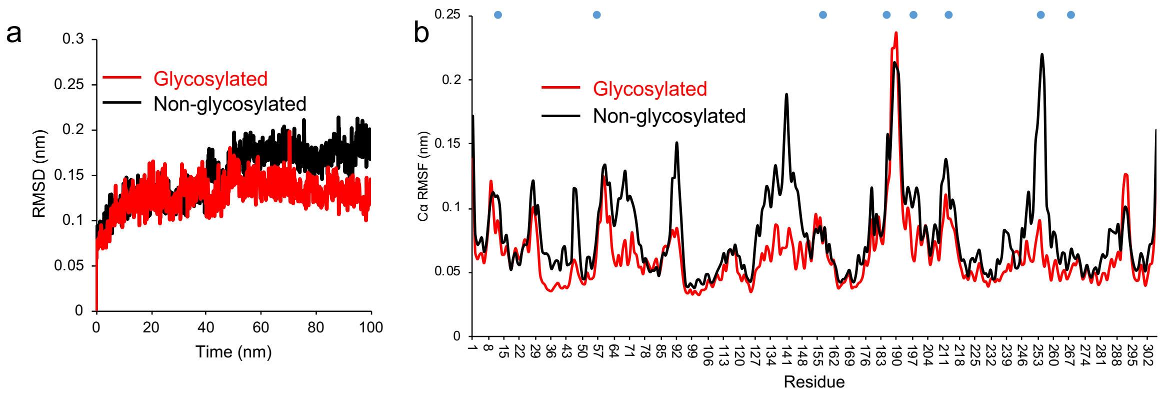

Figure S6. Molecular dynamics of glycosylated (red) and non-glycosylated (black) HRP. (a) Root mean squared deviation (RMSD) and (b) Ca root mean squared fluctuation over a 100 ns simulation. The blue circles represent the N-linked glycosylation sites.

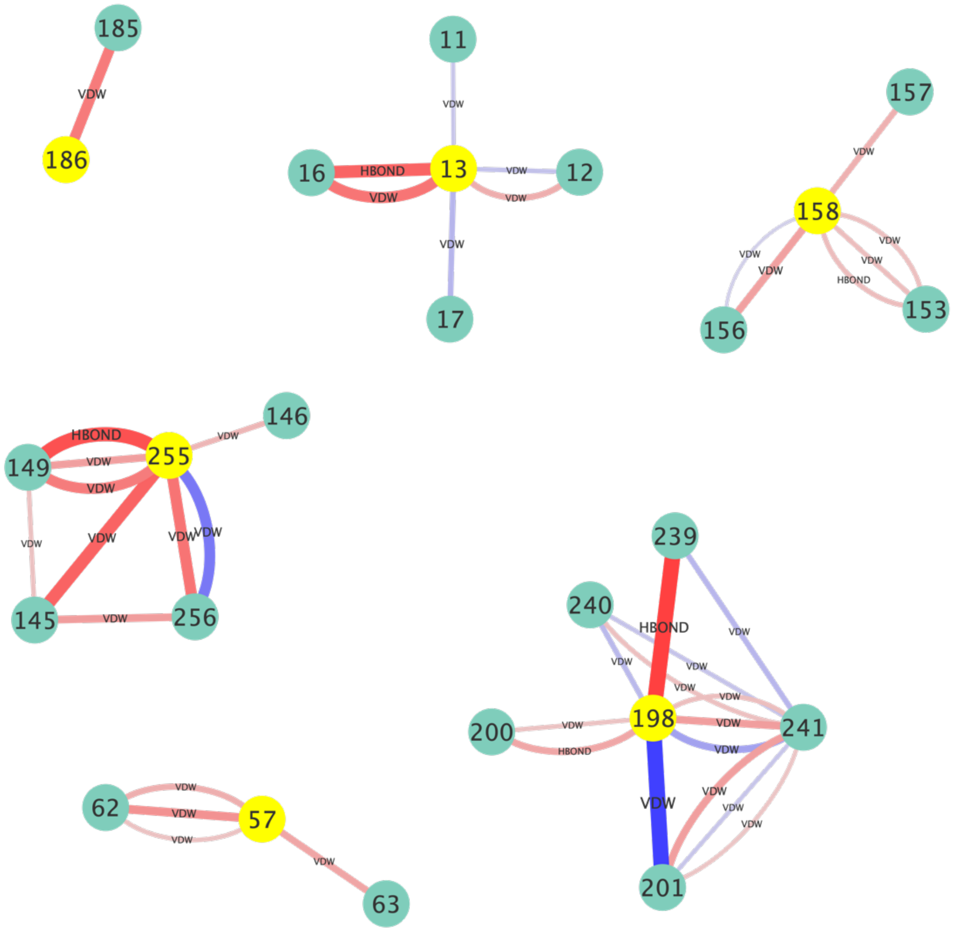

Figure S7. Change in pairwise interactions related to N-linked glycosylated Asn residues (yellow spheres) over the course of the MD simulation. Red lines indicate increased number of interactions in the glycosylated HRP and blue lines increased number of interactions in the non-glycosylated form. The thickness of the lines corresponds to the frequency of an interaction over the course of the MD, with thicker lines representing more persistent interactions. An arbitrary 10% cut off was applied, with interactions differences below this value ignored. The change in interaction type is shown on the diagram with VDW and HBOND equivalent to van der Waals and H-bonds, respectively.

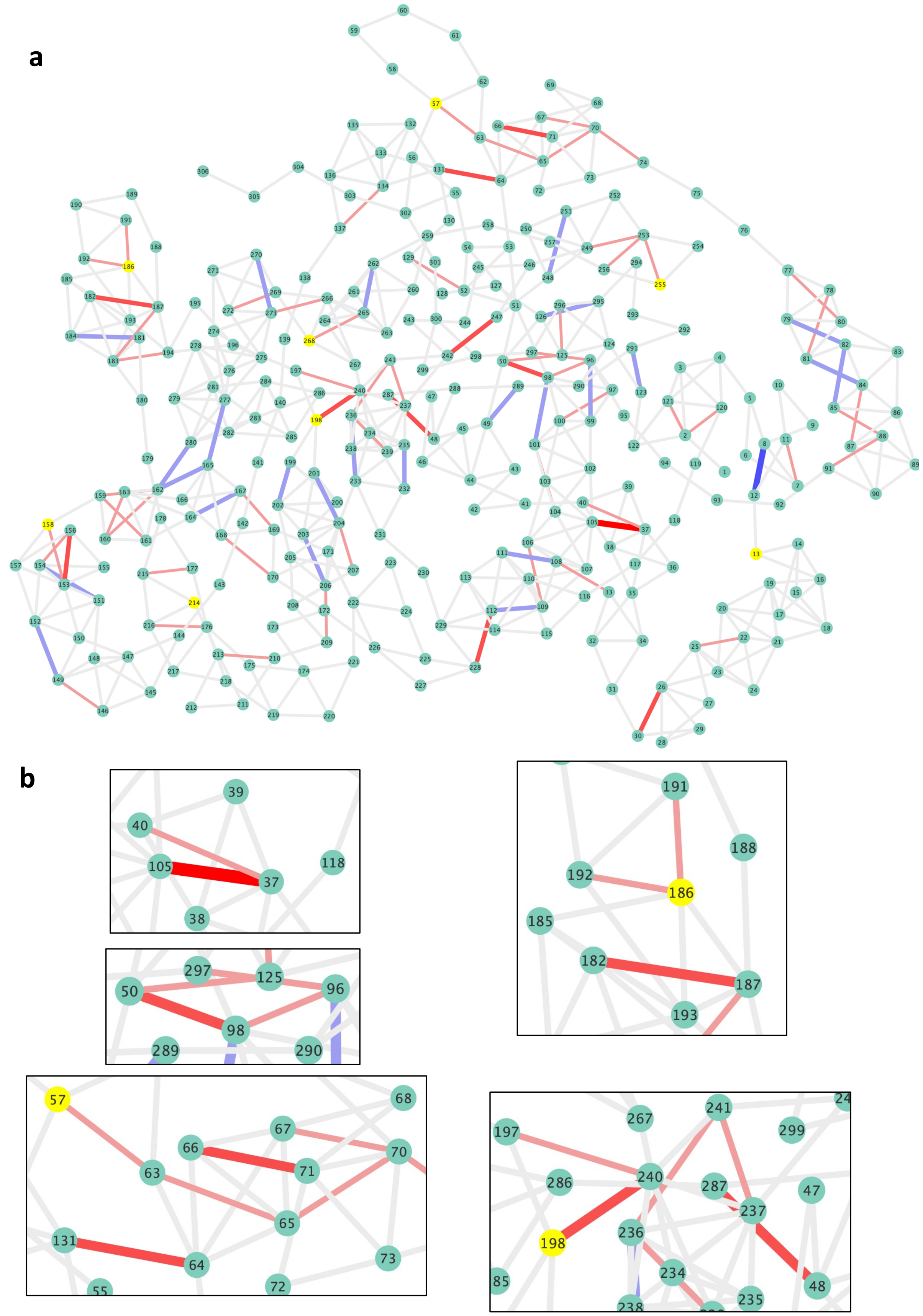

Figure S8. Pairwise network of Cα atoms with a 5.5Å cut-off over the course of the MD simulation. Grey links represent no change in the Cα pairwise distances with red lines indicating closer distances in the glycosylated form and blue lines indicating closer distances

in the non-glycosylated form with shade of colour and thickness of the line related to the change in distance (ranging from light colour thinner lines representing ~0.77Å to thicker darker red lines representing up to 1.15Å). (a) the whole pairwise network and (b) selected regions. N-linked glycosylation residues are shown a yellow spheres.

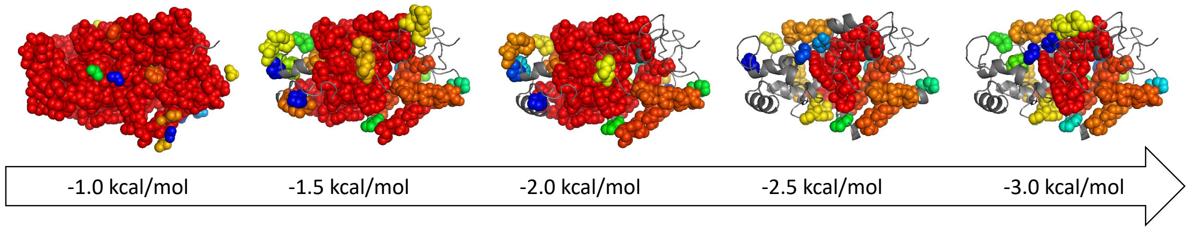

Figure S9. Rigid Cluster Decompositions (RCDs) of holo-HRP at different hydrogen-bond energy cut-offs in the course of a rigidity dilution. The twenty largest rigid clusters are shown in space-filling representation and rainbow-coloured from red to blue. Flexible regions are shown as grey cartoon. The largest rigid cluster, RC1 (red), spans almost the entire structure (at left) when the cut-off is small. Outlying portions of the structure become flexible as the cut-off becomes more negative during the dilution, until the largest rigid cluster includes only about twenty residues surrounding the haem group (at right). The dilution thus reveals the relative rigidity of different portions of the structure. Protein structure figures generated using PyMOL [6].

Cut off (kcal/mol)	RC1 value[a]			
	Holo	Apo	Holo+glycan	Apo+glycan
-0.5	299	299	299	299
-1.0	294	294	294	294
-1.5	126	61	180	142
-2.0	125	61	179	79
-2.5	23	22	23	25
-3.0	22	22	22	25
-3.5	16	16	25	25
-4.0	16	16	18	18

Table S1. Rigidity analysis of the different HRP forms. [a]RC1 value is the number of residues which are members of the largest rigid cluster. The size of the largest rigid cluster for holo- and apo-HRP with and without glycosylation is shown in the course of a rigidity dilution as the hydrogen-bond energy cutoff is lowered from -0.5 to -4.0 kcal/mol. All structures are almost entirely rigid at small cutoffs (-0.5, -1.0 kcal/mol) and mostly flexible at large cutoffs (-2.5 to -4.0 kcal/mol). Differences in the relative rigidity of the structures, due to the presence/absence of haem and glycosylation, are visible at intermediate cutoff values (-1.5, -2.0 kcal/mol).

References.

1. Fujiyama, K., Takemura, H., Shibayama, S., Kobayashi, K., Choi, J. K., Shinmyo, A., Takano, M., Yamada, Y. & Okada, H. (1988) Structure of the horseradish peroxidase isozyme C genes, *Eur J Biochem.* **173**, 681-7.
2. Wuhrer, M., Balog, C. I., Koeleman, C. A., Deelder, A. M. & Hokke, C. H. (2005) New features of site-specific horseradish peroxidase (HRP) glycosylation uncovered by nano-LC-MS with repeated ion-isolation/fragmentation cycles, *Biochim Biophys Acta.* **1723**, 229-39.
3. Kelly, J. F., Locke, S. J., Ramaley, L. & Thibault, P. (1996) Development of electrophoretic conditions for the characterization of protein glycoforms by capillary electrophoresis-electrospray mass spectrometry, *J Chromatogr A.* **720**, 409-27.
4. Welinder, K. G. (1979) Amino acid sequence studies of horseradish peroxidase. Amino and carboxyl termini, cyanogen bromide and tryptic fragments, the complete sequence, and some structural characteristics of horseradish peroxidase C, *Eur J Biochem.* **96**, 483-502.
5. Veitch, N. C. (2004) Horseradish peroxidase: a modern view of a classic enzyme, *Phytochemistry.* **65**, 249-59.
6. The PyMOL Molecular Graphics System version 2.5, Schrödinger, LLC,
